# Real World Outcomes versus Clinical Trial Results of Durvalumab Maintenance in Veterans with Stage III Non-Small Cell Lung Cancer

**DOI:** 10.3390/cancers14030614

**Published:** 2022-01-26

**Authors:** Kamya Sankar, Alex K. Bryant, Garth W. Strohbehn, Lili Zhao, David Elliott, Drew Moghanaki, Michael J. Kelley, Nithya Ramnath, Michael D. Green

**Affiliations:** 1 Division of Hematology and Oncology, Department of Internal Medicine, University of Michigan, Ann Arbor, MI 48109, USA; ksankar@med.umich.edu; 2 Rogel Cancer Center, University of Michigan, Ann Arbor, MI 48109, USA; bralex@med.umich.edu (A.K.B.); zhaolili@med.umich.edu (L.Z.); elliotda@med.umich.edu (D.E.); 3 Department of Radiation Oncology, University of Michigan, Ann Arbor, MI 48109, USA; 4 Section of Hematology and Oncology, Veterans Affairs Ann Arbor Healthcare System, Ann Arbor, MI 48105, USA; gstrohbe@med.umich.edu; 5 Department of Biostatistics, University of Michigan, Ann Arbor, MI 48109, USA; 6 Department of Radiation Oncology, Veterans Affairs Ann Arbor Healthcare System, Ann Arbor, MI 48105, USA; 7 Department of Radiation Oncology, UCLA Jonsson Cancer Center, Los Angeles, CA 90024, USA; dmoghanaki@mednet.ucla.edu; 8 Department of Veterans Affairs, Durham VA Medical Center, Durham, NC 27705, USA; kelleym@duke.edu; 9 Medical Oncology, Department of Medicine, Duke Medical Center, Durham, NC 27710, USA

**Keywords:** stage III non-small cell lung cancer, durvalumab, immunotherapy duration, veteran population, efficacy-effectiveness gap

## Abstract

**Simple Summary:**

The standard of care for patients with stage III non-small cell lung cancer is concurrent chemoradiotherapy followed by maintenance durvalumab based on outcomes from the PACIFIC trial. The efficacy of this regimen in a real-world population has not been extensively studied. We found that the addition of durvalumab has significantly improved both progression-free and overall survival in veterans with stage III non-small cell lung cancer as compared to veterans who received concurrent chemoradiotherapy alone, but overall survival of veterans is reduced compared to patients in the PACIFIC trial. Additional studies will need to be performed to understand this efficacy-effectiveness gap.

**Abstract:**

One year of durvalumab following concurrent chemoradiotherapy improves progression-free (PFS) and overall survival (OS) for patients with stage III non-small cell lung cancer (NSCLC). However, the real-world efficacy of durvalumab has not been determined. We conducted a multi-center observational cohort study across the Veterans Health Administration, including patients with stage III NSCLC who received concurrent chemoradiotherapy and durvalumab, compared to patients who received concurrent chemoradiotherapy alone. Kaplan–Meier and Cox regression approaches were used to identify factors associated with PFS and OS. We calculated a hazard ratio and efficacy-effectiveness factor to compare OS of veterans to the referenced clinical trial population. A total of 1006 patients with stage III NSCLC who received concurrent chemoradiotherapy and at least one dose of durvalumab from November 2017 to April 2021 were compared to 989 patients who received concurrent chemoradiotherapy alone from January 2015 to December 2016. Adjuvant durvalumab was associated with higher PFS (HR 0.62, 95% CI 0.55–0.70, *p* < 0.001) and OS (HR 0.57, 95% CI 0.50–0.66, *p* < 0.001). OS was shorter in veterans compared to PACIFIC (HR 1.24, 95% CI 1.03–1.48, *p* = 0.02: EE gap 0.73). OS of veterans with stage III NSCLC treated with adjuvant durvalumab is improved compared to a modern comparator but is reduced compared to the PACIFIC population.

## 1. Introduction

The PACIFIC trial [1] established 12 months of adjuvant durvalumab as the standard of care for patients with stage III non-small cell lung cancer (NSCLC) after definitive concurrent chemoradiotherapy (cCRT). Updated analyses with a median follow up of 34 months demonstrated a 9.5% absolute 5-year overall survival benefit with the addition of maintenance durvalumab [2]. The improvement in survival was noted with an acceptable toxicity profile and without compromise in patient-reported outcomes [3]. However, the effect size of adjuvant durvalumab has not been measured extensively with real world evidence, and there may be a gap between the efficacy of durvalumab demonstrated in PACIFIC and its effectiveness in clinical practice.

Though adjuvant durvalumab for 12 months after cCRT has been the standard of care for eligible veterans across Veterans Health Administration (VHA) hospitals nationwide since the introduction of durvalumab, veterans receiving care within the VHA were not included in the PACIFIC trial. Veterans represent a patient population characterized by significant medical co-morbidities and tobacco exposure, which may impact tolerance to oncologic therapies. The objectives of this study were to examine treatment adherence, toxicity, and oncologic outcomes in veterans with stage III NSCLC, treated with curative intent cCRT with or without durvalumab consolidation. Additionally, we correlate the clinical outcomes of veterans with stage III NSCLC treated with adjuvant durvalumab (effectiveness) to outcomes reported in the PACIFIC trial (efficacy), using data from the largest integrated healthcare system in the United States.

## 2. Materials and Methods

### 2.1. Data Source

We identified lung cancer patients using the Department of Veterans Affairs (VA) Informatics and Computing Infrastructure (VINCI). VINCI is an informatics platform that allows access to patient-level electronic health record information and administrative data for all veterans within the VA healthcare system. VINCI also incorporates tumor registry data uploaded from individual VA sites; these data are gathered by trained registrars according to standard protocols. This study was approved by the local institutional review board.

### 2.2. Patient Selection

We included consecutive patients with histologically confirmed stage III NSCLC (AJCC 8th edition) treated with cCRT and at least one dose of adjuvant durvalumab between November 2017 to April 2021 (Cohort 1). The first and last durvalumab infusion dates were first identified with outpatient infusion records and confirmed by manual chart review for all patients in Cohort 1. Staging and definitive treatment information were subsequently obtained by manual review of the medical record. These staging and treatment data were supplemented with data from the Veterans Affairs Cancer Registry System (VACRS) where available. For historical comparison of oncologic outcomes, we identified a cohort of stage III NSCLC patients treated consecutively with cCRT alone between January 2015 and December 2016 (Cohort 2). These patients were identified through treatment and staging records in the VACRS.

### 2.3. Outcomes and Covariates

The primary outcome measures were progression-free survival (PFS) and overall survival (OS). Date of radiographic progression was determined and confirmed by manual review of radiological reports by a licensed physician (M.D.G. and K.S.). Date of death was obtained from the VA Vital Status File (drawn from Medicare, Social Security Administration, and the internal VA death registry; available for 81% of the cohort) and supplemented with the VA Master Patient Index for more recent deaths (19%).

Demographics, including race, sex, and age, were obtained through the Master Patient Index. Charlson Comorbidity Index (CCI) [4,5] was calculated from inpatient and outpatient ICD-10 diagnosis codes in the year before durvalumab start (Cohort 1) or the proxy durvalumab start date described below (Cohort 2). Smoking status was obtained through Health Factors data [6,7]. A concurrent chemotherapy regimen was obtained through intravenous infusion records and supplemented with the VACRS where available. Durvalumab treatment duration was defined as the difference in days between the first and most recent infusion dates; this was defined as 1 day for patients with a single infusion. The number of durvalumab infusions and reason for durvalumab discontinuation (classified as progression, immune-related adverse event [irAE], non-irAE toxicity, declining performance status, patient preference, lost-to-follow-up, death, or other/unknown) were obtained through manual review of physician notes. Patients were categorized as having durvalumab-related toxicity if the toxicity was possibly, probably, or definitely related to durvalumab in the judgement of the managing outpatient oncologist or inpatient physician.

### 2.4. Statistical Analysis

Differences in baseline characteristics were assessed with the chi-square test for categorical variables and the t-test for continuous variables. OS and PFS estimates were generated with the Kaplan–Meier method and were compared between Cohort 1 and Cohort 2 with the log-rank test in univariable analyses. Adjusted survival analyses between Cohorts 1 and 2 were performed with multivariable Cox regression, adjusting for age (continuous, per 10 years), sex (male vs. female), race (African American, Caucasian, or other/unknown), smoking status (current, former, never, or unknown), CCI (0–2, 3–5, 6–8, or ≥9), AJCC stage (IIIA, IIIB, IIIC, or III not otherwise specified), concurrent chemotherapy regimen (carboplatin-paclitaxel vs. other), and histology (adenocarcinoma, squamous cell carcinoma, or other). In Cohort 1, survival time was measured from the first dose of durvalumab to death from any cause (for OS), or to disease progression or death from any cause (for PFS). In Cohort 2, as there was no durvalumab start date, this date was proxied as the date of radiation start plus 86 days (the median time from radiation start to durvalumab start in Cohort 1) and survival time was calculated as in Cohort 1. To mitigate selection bias, as all patients in Cohort 1 were, by definition, eligible for durvalumab consolidation, patients in Cohort 2 who progressed prior to the imputed durvalumab start date were excluded (*n* = 48). Patients were censored at the date of last known follow-up, defined as the most recent encounter with a VA provider. Patients with ongoing follow-up past 15 April 2021, were administratively censored at that time.

The efficacy-effectiveness gap was assessed using two methods. First, an efficacy-effectiveness (EE) factor was calculated by dividing each cohort’s median overall survival by the corresponding reference OS from the most recent report from PACIFIC [1,8,9]. This factor was used to estimate the presence of an EE gap and compare the real-world population’s survival relative to the clinical trial population. As an example, an EE factor of 0.60 indicates that median survival is 40% shorter in clinical practice than in the reference clinical trial. Second, we used hazard ratios between real-world cohorts and the clinical trial cohorts to compare PFS and OS. This was achieved by reconstructing individual patient data from the Kaplan–Meier curves from PACIFIC with an online tool and incorporating reconstructed patient data into proportional hazards regression models, including both VA and reconstructed PACIFIC data [10]. All statistical analyses were performed using SAS 9.4 (SAS Institute Inc, Cary, NC, USA) and R v4.0.2 (R Core Team, Vienna, Austria).

## 3. Results

### 3.1. Patient Characteristics

We identified 1006 patients with stage III NSCLC who received cCRT followed by at least one dose of durvalumab (Cohort 1) and 989 patients who received cCRT alone in the pre-durvalumab comparison cohort (Cohort 2). Among all patients, the median age was 68 years (interquartile range [IQR]: 64 to 72) and the majority were male (96.3%) and Caucasian (75.8%). Most patients were current (43.5%) or former (36.9%) smokers. A total of 41.6% had adenocarcinoma histology and 50.5% had squamous cell histology. Patients in Cohort 2 had lower rates of severe comorbidity (26.1% with CCI 9 or higher vs. 37.7% in Cohort 1) and higher rates of stage IIIA disease (67.4% vs. 55.6% in Cohort 1) but were otherwise similar to Cohort 1 in other baseline covariates. The patient characteristics of both Cohorts 1 and 2 in relation to the patients included in the PACIFIC trial are shown in Table 1.

### 3.2. Progression-Free and Overall Survival

**Figure 1:**
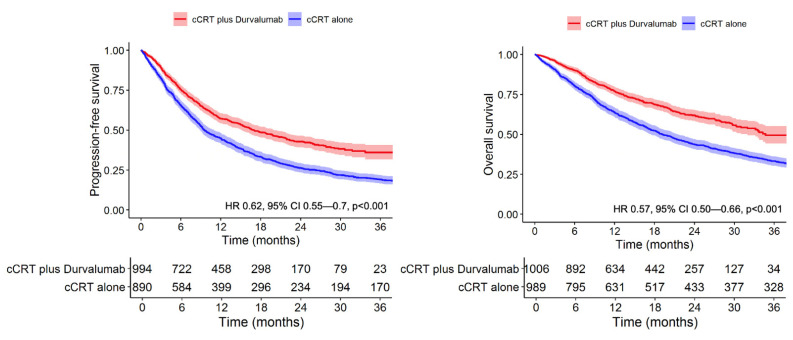
Adjuvant durvalumab significantly extends progression-free and overall survival in Veterans with stage III NSCLC.

Patients in Cohort 1 showed higher rates of PFS and OS compared to Cohort 2 at all time-points examined (*p* < 0.001 by log-rank for both comparisons) (Figure 1). The median follow-up among censored patients was 19.9 months (Cohort 1) and 58.4 months (Cohort 2). Unadjusted 12-month and 24-month PFS was 57.2% (95% CI 54.0–59.7) and 42.7% (95% CI 39.2–46.3) in Cohort 1 compared to 44.9% (95% CI 41.6–48.2) and 26.3% (95% CI 23.4–29.2) in Cohort 2. The median PFS was 16.9 months (95% CI 17.1–20.3) in Cohort 1 versus 9.6 months (95% CI 9–11.1) in Cohort 2. The median OS was 34.7 months (95% CI 31.5-NR) in Cohort 1 versus 19.2 months (95% CI 17.6–21.6) in Cohort 2. Unadjusted 12-month and 24-month OS was 77.0% (95% CI 74.4–79.7) and 61.9% (95% CI 58.4–65.3) in Cohort 1 compared to 63.9% (95% CI 60.9–66.9) and 43.8% (95% CI 40.7–46.9) in Cohort 2.

In multivariable analysis after adjustment for confounders, Cohort 1 showed improved PFS (adjusted HR 0.62, 95% CI 0.55–0.70, *p* < 0.001) and OS (adjusted HR 0.57, 95% CI 0.50–0.66, *p* < 0.001) compared to Cohort 2 (Table 2). In the model for OS, the strongest predictors of death included increasing age (HR 1.20 per 10 years, 95% CI 1.10–1.32, *p* < 0.001) and higher CCI (HR 1.26 for 9+ vs. 0–2, 95% CI 1.06–1.5, *p* = 0.008). In the model for PFS, predictors for shorter PFS included male sex (HR 1.36, 95% CI 0.95–1.94, *p* = 0.09), advancing age (HR 1.12 per 10 years, 95% CI 1.03–1.22, *p* = 0.009), and stage IIIC disease (HR 1.49, 95% CI 1.07–2.07, *p* = 0.019). Squamous cell histology was associated with improved PFS (HR 0.86, 95% CI 0.77–0.97, *p* = 0.01).

### 3.3. Efficacy-Effectiveness Factor Analysis

**Figure 2:**
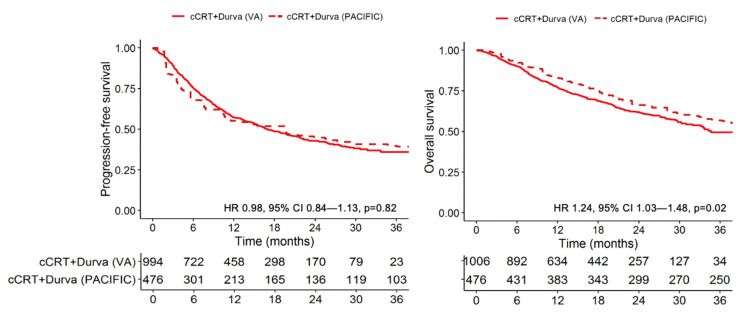
Kaplan–Meier progression-free and overall survival curves of real-world and clinical trial patients with stage III NSCLC who receive concurrent chemoradiotherapy and durvalumab.

To compare the real-world and clinical trial survival outcome of patients who received cCRT and durvalumab, we calculated the efficacy-effectiveness factor and hazard ratio for OS, comparing Cohort 1 to the durvalumab group in PACIFIC. Veterans who received cCRT plus durvalumab (Cohort 1) had an EE factor of 0.73, indicating that median survival was 27% shorter for patients treated in clinical practice relative to median survival from the registered clinical trial receiving the same treatment; the corresponding HR was 1.24 (95% CI 1.03–1.48, *p* = 0.02) (Figure 2). There was no significant difference in PFS (HR 0.98, 95% CI 0.84–1.13, *p* = 0.82) (Figure 2).

When investigating explanatory factors for why veterans had a shorter OS, we identified that veterans in Cohort 1 received shorter duration of durvalumab therapy than patients in PACIFIC with a higher incidence of treatment discontinuation for toxicity. Among patients in Cohort 1, the median number of durvalumab infusions was 12 (IQR: 5 to 23) and the median duration of durvalumab therapy was 215 days (IQR: 84 to 350). In comparison, patients in the PACIFIC trial received a median of 20 infusions of durvalumab (range, 1 to 27) for a median duration of 310 days of treatment. The most common reasons for durvalumab discontinuation in Cohort 1 were completion of planned therapy (*n* = 314, 31.2%), disease progression (*n* = 221, 21.9%), irAE (*n* = 152, 15.1%), and non-irAE toxicity attributed to durvalumab (n = 60, 5.9%). Among the 152 patients who discontinued durvalumab due to irAE, the most common grade 3 or higher irAE event was pneumonitis (*n* = 109, 10.8%). A total of 136 patients (13.5%) had ongoing durvalumab therapy at the time of last follow-up. In the PACIFIC cohort, among 713 patients, reasons for durvalumab termination included completion of therapy (*n* = 202, 28.3%), disease progression (n = 148, 20.8%), and adverse event leading to treatment discontinuation (*n* = 73, 10.2%). The incidence of grade 3 or 4 pneumonitis in PACIFIC was 3.4%.

## 4. Discussion

We report the largest series of patients with stage III NSCLC treated with cCRT and adjuvant durvalumab in a cohort of over 1000 veterans who received their care at a VHA medical center. We show that durvalumab consolidation in veterans is associated with a large PFS and OS benefit relative to contemporary historical controls, though OS in the veteran population is still inferior to durvalumab-treated patients in PACIFIC, with an EE gap of 0.73. To our knowledge, this is the first study that directly compares real-world and clinical trial cohorts within a population of patients with stage III NSCLC who were not included in the PACIFIC trial. An observational cohort multicenter international study to evaluate real-world efficacy of cCRT and adjuvant durvalumab in patients with stage III NSCLC is ongoing but data have not yet been reported (PACIFIC-R, NCT03798535). Furthermore, the veteran population is diverse, including patients with co-morbidities, older age, and those living in rural areas, who are likely to be excluded from many clinical trials. Thus, these data represent a unique means for assessing real-world outcomes for this important new therapy.

The results demonstrate that the use of durvalumab consolidation among veterans after cCRT is associated with a significant improvement in both OS and PFS compared to a modern cohort treated without durvalumab. Our estimates of OS and PFS in Cohort 2 are similar to previously published historical cohorts of veterans who received cCRT alone [11]. Reassuringly, we show that PFS of patients with stage III NSCLC treated with cCRT and adjuvant durvalumab was comparable between real-world and clinical trial populations (median PFS 16.9 vs. 16.8 months). Despite the similar PFS, OS was significantly shorter for veterans who received adjuvant durvalumab (median OS 34.7 vs. 47.5 months in PACIFIC, EE gap of 0.73), potentially implicating a higher rate of competing mortality in the veteran population or inferior treatment tolerance leading to mortality. Veterans received a shorter median duration of durvalumab therapy as compared to PACIFIC (7.1 vs. 10.0 months) and had a higher rate of durvalumab discontinuation due to toxicity (21% vs. 15%), particularly pneumonitis (10.8% vs. 3.4% Grade 3–4 pneumonitis in PACIFIC). Given the higher rate of therapy discontinuation due to toxicity, prospective evaluations of optimal treatment duration of adjuvant durvalumab therapy to balance efficacy and toxicity in this population may be warranted.

As with other real-world studies of immune checkpoint inhibitor use [12,13,14,15,16,17], overall survival outcomes tended to be shorter than those observed in the reference clinical trial. Several factors may have contributed to shorter overall survival in the VA cohort, including patient characteristics of the veteran population as well as potential bias and confounding arising from the use of CDW data in a retrospective analysis. The veteran cohort is predominantly male, has a higher degree of comorbidities and is older than populations represented in clinical trials. Another striking difference in our cohort is the higher percentage of durvalumab discontinuation for toxicity. Though previous studies have noted a positive association between development of irAE and PFS, others have shown that grade 3 or 4 irAEs have been associated with worse OS, due to a higher incidence of treatment-related mortality [18]. Pneumonitis represents the potentially most severe and life-threatening of all reported immunotherapy-related adverse events and is further complicated in the context of prior therapies also known to cause pulmonary toxicity, such as radiotherapy. We report a higher incidence of pneumonitis severe enough to warrant durvalumab discontinuation in our cohort as compared to PACIFIC. Veterans have been shown to have higher prevalence of tobacco use [19,20] and chronic obstructive pulmonary disease as compared to the general population [21,22], both of which have been implicated as a risk factor for the development of immune mediated pneumonitis in retrospective analyses [23]. Patients with ongoing exposure to cigarette smoke in a setting of radiotherapy may result in a higher risk of developing drug-related lung toxic effects [24]. A significantly higher proportion of patients in our Cohort treated with durvalumab were current smokers as compared to the population treated with durvalumab in the PACIFIC trial (43.2% vs. 16.6%). Furthermore, smoking and baseline lung disease have also been correlated with higher grade pneumonitis that is refractory to steroid therapy [25,26,27].

Strengths of our analysis include large patient numbers, contemporary time period, a modern comparison cohort, and the availability of patient charts to manually confirm treatment dates and ascertain reasons for durvalumab discontinuation within an integrated healthcare system. Methods to ascertain staging in both cohorts were similar, using manual chart review and supplementation with data from the VACRS where applicable. Stage migration in patients with NSCLC through widespread adoption of fluorodeoxyglucose-positron emission tomography [28,29] has not been shown to cause significant changes in stage migration after the year 2002 [30], thereby not affecting our comparison cohort of patients treated from 2015 to 2016. Limitations of our analysis include the differential methods for ascertaining patients who received durvalumab (Cohort 1) and patients who would have been eligible to receive durvalumab (Cohort 2), which may have resulted in an optimistic selection bias in favor of Cohort 1. To mitigate this, we excluded patients in Cohort 2 who progressed prior to the imputed durvalumab start date. Reassuringly, we did not find evidence of substantial differences between groups in measured covariates; in fact, the durvalumab group had somewhat higher rates of severe comorbidity compared to the comparator group, opposite to the expected finding in the presence of healthy-user bias. The comparison of durvalumab-treated patients and historical comparators is further subject to the usual limitations of retrospective treatment comparisons, including the presence of unmeasured confounding and unrecognized selection bias.

## 5. Conclusions

Veterans with stage III NSCLC treated with curative intent cCRT and adjuvant durvalumab have significant improvement in progression-free and overall survival compared to patients who received cCRT alone in a modern comparison veteran cohort and historical data. Despite similar PFS rates, OS is reduced in the veteran population compared to the PACIFIC trial population. Further investigations are warranted to identify factors which may lead to higher rates of durvalumab discontinuation, higher incidence of pneumonitis, and shorter overall survival in veterans with stage III NSCLC who are treated with cCRT and adjuvant durvalumab.

## Figures and Tables

**Table 1:** Characteristics of patients who received cCRT plus durvalumab versus cCRT alone in the real-world setting and in the PACIFIC trial.

Variable		Cohort 1 (cCRT Plus Durvalumab)	Cohort 2 (cCRT Alone)	*p*-Value *	Durvalumab Group (PACIFIC) [1]	Placebo Group (PACIFIC) [1]
*N*		1006	989		476	237
Age, median in years (IQR)		69 (64–72)	68 (64–71)	0.009	64	64
Race, *n* (%)	African American	221 (22.0)	161 (16.3)	0.001	120 (25.2)	72 (30.4)
	Caucasian	745 (74.1)	767 (77.6)		337 (70.8)	157 (66.2)
	Other/unknown	40 (3.98)	61 (6.17)		120 (25.2)	72 (30.4)
Sex, *n* (%)	Female	47 (4.67)	26 (2.63)	0.015	142 (29.8)	71 (30.0)
	Male	959 (95.3)	963 (97.4)		334 (70.2)	166 (70.0)
CCI, *n* (%)	0–2	148 (14.7)	241 (24.4)	<0.001		
	3–5	342 (34.0)	363 (36.7)			
	6–8	137 (13.6)	127 (12.8)			
	9+	379 (37.7)	258 (26.1)			
Smoking, *n* (%)	Current	435 (43.2)	432 (43.7)	0.001	79 (16.6)	38 (16.0)
	Former	402 (40.0)	334 (33.8)		354 (74.4)	178 (75.1)
	Never	87 (8.65)	98 (9.91)		43 (9.0)	21 (8.9)
	Unknown	82 (8.15)	125 (12.6)		-	-
Stage, *n* (%)	IIIA	559 (55.6)	667 (67.4)	<0.001	252 (52.9)	125 (52.7)
	IIIB	352 (35.0)	322 (32.6)		212 (44.5)	107 (45.1)
	IIIC	66 (6.56)	--		-	-
	III NOS	29 (2.88)	--		12 (2.5)	5 (2.1)
Concurrent chemotherapy, *n* (%)	Carboplatin/paclitaxel	711 (70.7)	700 (70.8)	<0.001		
	Cisplatin/etoposide	62 (6.16)	92 (9.30)			
	Platinum/pemetrexed	106 (10.5)	6 (0.61)			
	Other/unknown	127 (12.6)	191 (19.3)			
Histology	Adenocarcinoma	490 (48.7)	340 (34.4)	<0.001	252 (52.9)	135 (57.0)
	Squamous cell carcinoma	485 (48.2)	522 (52.8)		224 (47.1)	102 (43.0)
	Other	31 (3.08)	127 (12.8)		-	-
Time from RT end to durvalumab start, median in days (IQR)		42 (29–63)	--			

* *p*-Value represents a comparison in baseline characteristics between Cohorts 1 and 2.

**Table 2:** Multivariable Cox regression analysis of progression-free survival and overall survival in veterans with stage III NSCLC.

		PFS		OS	
Variable		HR (95% CI)	*p*-Value	HR (95% CI)	*p*-Value
Cohort	Cohort 2 (pre-durvalumab)	Ref	Ref	Ref	Ref
	Cohort 1 (durvalumab)	0.62 (0.55–0.70)	<0.001	0.57 (0.50–0.66)	<0.001
Age (per 10 years)		1.12 (1.03–1.22)	0.009	1.20 (1.10–1.32)	<0.001
Male		1.36 (0.95–1.94)	0.09	1.33 (0.92–1.93)	0.13
Race	African American	Ref	Ref	Ref	Ref
	Caucasian	1.03 (0.89–1.19)	0.69	1.16 (0.98–1.36)	0.08
	Other/unknown	1.05 (0.81–1.37)	0.71	1.05 (0.78–1.41)	0.74
Smoking	Current	Ref	Ref	Ref	Ref
	Former	1.01 (0.89–1.15)	0.85	1.04 (0.91–1.19)	0.58
	Never	1.03 (0.85–1.25)	0.79	1.01 (0.82–1.24)	0.94
	Unknown	1.07 (0.89–1.30)	0.47	1.10 (0.90–1.34)	0.34
Stage	IIIA	Ref	Ref	Ref	Ref
	IIIB	1.23 (1.09–1.38)	<0.001	1.21 (1.07–1.37)	0.003
	IIIC	1.49 (1.07–2.07)	0.019	1.23 (0.81–1.86)	0.32
	III NOS	0.89 (0.51–1.57)	0.69	0.95 (0.48–1.85)	0.87
Chemotherapy	Other/unknown	Ref	Ref	Ref	Ref
	Carboplatin/paclitaxel	1.01 (0.89–1.14)	0.93	0.96 (0.85–1.10)	0.58
Histology	Adenocarcinoma	Ref	Ref	Ref	Ref
	Squamous cell carcinoma	0.86 (0.77–0.97)	0.01	0.97 (0.85–1.10)	0.59
	Other	1.02 (0.83–1.25)	0.87	1.07 (0.86–1.32)	0.55
CCI	0–2	Ref	Ref	Ref	Ref
	3–5	1.18 (1.01–1.38)	0.04	1.22 (1.03–1.43)	0.02
	6–8	1.30 (1.07–1.58)	0.008	1.12 (0.91–1.38)	0.30
	9+	1.20 (1.02–1.41)	0.03	1.26 (1.06–1.50)	0.008

## Data Availability

The data presented in this study are available in this paper.
